# Supplementary material for: Does quality of antenatal care influence antepartum stillbirth in Hossana City, South Ethiopia?
Source: PLOS Glob Public Health. 2023 Jan 26;3(1):e0001468. doi: 10.1371/journal.pgph.0001468 (PMC10021135; doi:10.1371/journal.pgph.0001468)
Supplement: Attachment — Submitted filename: Response to reviewers.docx [file pgph.0001468.s001.docx]

**Point by point response letter**

| S/n | Comment By the Editor | Response | Remark |
| --- | --- | --- | --- |
| 1 | Please amend your detailed online Financial Disclosure statement | Comment accepted and included like this  **Funding (financial disclosure statement)**  TTB received the grant from Yekatit 12 Hospital Medical College, Addis Ababa, Ethiopia.. The funder (Yekatit 12 Hospital Medical College) covers only the transport and per diem cost of data collectors and supervisors during data collection process. The funders had no role in study design, analysis, decision to publish, or preparation of the manuscript except covering the afore mentioned costs during data collection period | See the track change |
| 2 | Please update your online Competing Interests statement. If you have no competing interests to declare, please state: “The authors have declared that no competing interests exist.” | Comment accepted and corrected like this  The authors have declared that no competing interests exist | See the track change |
| 3 | Please provide separate figure files in .tif or .eps format and remove any figures embedded in your manuscript file. Please also ensure that all files are under our size limit of 10MB. | Comment accepted and corrected  in the form of TIFF . And in separate form. |  |
| 4 | We suggest you thoroughly copyedit your manuscript for language usage, spelling, and grammar | Thank you comment accepted and corrected | See the track change |
| Response to reviewer 1 | | | |
| 1 | I think the title has some few issues. For example “influence” is wrongly spelled. I also think that the ‘?’ sign has been placed at the wrong place. If you need to include it in the title at all, then it could come at the end. | Thank you comment accepted and amended accordingly | See the track change |
| 2 | Abstract The journal requires that the abstract is structured. I can see the authors have attempted to do that but the “Background” heading is missing. | Thank you comment accepted and amended accordingly | See the track change |
| 3 | The odds of poor quality of ANC services among pregnant mothers 0.19 (AOR 0.19) at 95% CI; 0.088 to 0.435). Thus, those mothers who received poor quality of ANC services are 81% more likely to develop antepartum still birth compared to those mothers who received good quality of ANC services”. | Comment accepted and amended accordingly | see the track change |
| 4 | The abstract does not even tell readers about the percentage of poor quality ANC recorded in the study. | Comment accepted and amended accordingly | see the track change |
| 3 | I think it's unfortunate that the authors believe this. In general, in SSA, there is a disconnect between the use of ANC and delivery at health facilities; women use ANC but deliver at home. Although the use of ANC enhances the likelihood of delivery in a health facility, this is not always the case. Many factors, not simply ANC, influence the location of delivery. Some elements, such as the commencement of labour and delivery, are out of the individual's control. I'm not convinced the authors aim to draw the conclusion that all women who give birth in health facilities get good ANC and all women who give birth at home get bad ANC. The sentence should be removed or altered, in my opinion. | Although one of the main components of focused ANC is birth preparedness and complication readiness which is thought to promote institutional delivery , unfortunately this not happening in Ethiopia .Due to this reason We considered those mothers who gave birth at home as if they were experiencing antepartum still birth .  But we couldn’t ignore your comment and concerns since in the limitation of the study was stated as one of the main concern was over estimation of antepartum still birth. |  |
|  | Data analysis. Based on Hosmer and Lemeshow applied logistic regression guide a p-value < 0.2 was considered to select… Setting criteria for variable exclusion in an adjusted model is intended to ensure model stability, especially when dealing with a large number of exposure variables. Although it has apparent advantages, one drawback of this technique is that significant variables are "thrown away" simply because they do not fulfil the inclusion criteria. One thing to keep in mind when working with datasets is that statistical significance is determined in part by sample size. Because there are few | Thank you for making our manuscript more valuable and it has been already incorporated. |  |
|  | What were the ethical considerations in the current study? | Thank you for making our manuscript more valuable and it has been already incorporated.  This study was a component of Strategies for quality antenatal care to improve birth outcome, which received institutional review board approval from University of south Africa(UNISA reference number (UNISA-ET/KA/ST/29/14-06-17).), and from Southern nations and nationalities people’s region Ethiopian Health Bureaus and Hadiya Zone. Both ANC clients and providers were informed about the purpose of the study and verbal and written informed consent was also obtained before data collection by explaining the goals of the study, the credibility of the researchers’ confidentiality, anonymity and the freedom to opt out of the study at any stage without negative consequences. Furthermore, report writing didn’t refer a specific respondent with identifiers | see the track change |
|  | The descriptive information is problematic and with many grammar problems. Authors need to check again. For instance what do they mean by “Pregnancy greater than 34”. I don’t see anything like that in Table 1. Also, some of the percentages (e.g. Age variable) do not sum up to 100. Is the age in years or year? | Amended as per the recommendation | see track change |
|  | “Almost half 445 (45.4%) were attempted abortion at least 1 times (Figure 1)”… This is not standard English.  What is the relevance of the “S/N” in the tables? Why does Table 2 start from S/N 11 before 2? | Amended as per the recommendation |  |
|  | Define the abbreviations used in the tables | Comment accepted and incorporated in list of abbreviations as well as in the table |  |
|  | Limitation I'm not sure why the writers' statement is considered a limitation. When women who deliver outside of a health facility are included, the coverage is broader than when only women who birth in a health facility are included. | This comment is already addressed in comment number three. |  |
|  | Discussion and conclusion I expect these two sections to change based on how the main results are interpreted. | Amended as per the recommendation | See the track change |
